# *Escherichia coli* from biopsies differ in virulence genes between patients with colorectal neoplasia and healthy controls

**DOI:** 10.3389/fmicb.2023.1141619

**Published:** 2023-04-13

**Authors:** Juraj Bosák, Darina Kohoutová, Matěj Hrala, Jitka Křenová, Paula Morávková, Stanislav Rejchrt, Jan Bureš, David Šmajs

**Affiliations:** ^1^Department of Biology, Faculty of Medicine, Masaryk University, Brno, Czechia; ^2^Center of Biomedical Research, University Hospital Hradec Králové, Hradec Králové, Czechia; ^3^The Royal Marsden Hospital NHS Foundation Trust, London, United Kingdom; ^4^Second Department of Internal Medicine - Gastroenterology, Charles University, Faculty of Medicine in Hradec Králové, University Hospital Hradec Králové, Hradec Králové, Czechia

**Keywords:** *Escherichia coli*, virulence factors, colorectal neoplasia, cancer, genotoxin, invasion, *ibeA*

## Abstract

**Introduction:**

Pathogenic strains of *Escherichia coli* have been clearly identified as the causative agents of extraintestinal and diarrheal infections; however, the etiopathogenic role of *E. coli* in other conditions, including colorectal cancer, remains unclear.

**Methods:**

This study aimed to characterize mucosal *E. coli* isolates (*n* = 246) from 61 neoplasia patients and 20 healthy controls for the presence of 35 genetic determinants encoding known virulence factors.

**Results:**

Virulence determinants encoding invasin (*ibeA*), siderophore receptor (*iroN*), S-fimbriae (*sfa*), and genotoxin (*usp*) were more prevalent among *E. coli* isolated from patients with neoplasia compared to the control group (*p* < 0.05). In addition, the prevalence of these virulence determinants was increased in more advanced neoplasia stages (*p*_adj_ < 0.0125). Compared to patients with advanced colorectal adenoma and carcinoma, the *ibeA* gene was rarely found in the control group and among patients with non-advanced adenoma (*p* < 0.05), indicating its potential as the advanced-neoplasia biomarker. Patients with neoplasia frequently had *E. coli* strains with at least one of the abovementioned virulence factors, whereby specific combinations of these virulence factors were found.

**Discussion:**

These findings suggest that *E. coli* strains isolated from patients with colorectal neoplasia possess several virulence factors, which could contribute to the development of neoplastic processes in the large intestine.

## Graphical Abstract

This study found a positive association between four virulence-associated genes (VAGs) of *E. coli* and colorectal neoplasia. These VAGs were predominantly found among *E. coli* from patients with advanced adenoma or carcinoma. Presence of certain *E. coli* in the gut appears to be a cause rather than a consequence of colorectal neoplasia. Created with BioRender.com. 
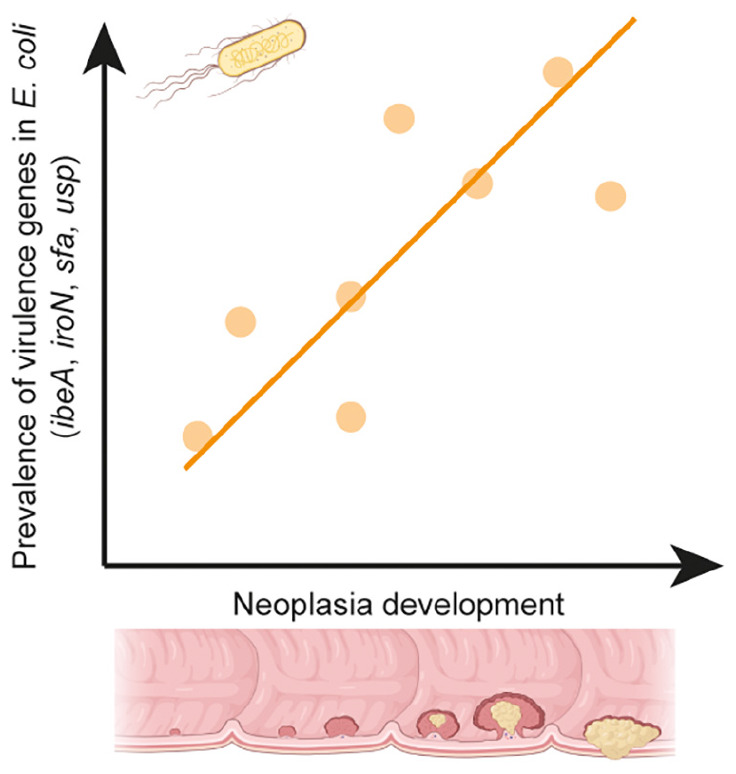

## Highlights

– This study found a positive association between several *E. coli* virulence-associated genes and colorectal neoplasia.
– Compared to healthy controls, mucosal *E. coli* from neoplasia patients more frequently encoded invasins, adhesins, and genotoxins, especially in cohorts of patients with advanced neoplasia and colorectal cancer.
– The gene for invasin (*ibeA*) has potential to be a one of the neoplasia biomarkers, since it was found almost exclusively among patients with advanced colorectal adenoma and carcinoma.
– The fact that *E. coli* strains encode the same virulence factors in subsets of patients with current and previous neoplasia indicates that the identified *E. coli* strains appear to contribute to the development of colorectal neoplasia, as opposed to being a consequence of neoplastic conditions.

## Introduction

*Escherichia coli* is a commensal bacterium of the human gastrointestinal tract and, at the same time, an important human pathogen. Pathogenic *E. coli* emerged from non-pathogenic strains by the acquisition of virulence factors. Based on encoded virulence factors, they can be classified as extraintestinal and intestinal pathogenic *E. coli* (Nataro and Kaper, 1998; Kaper et al., 2004; Tenaillon et al., 2010; Riley, 2020; Denamur et al., 2021).

Extraintestinal pathogenic *E. coli* (ExPEC) strains colonize various sites of the human body and are associated with a spectrum of infections ranging from uncomplicated urinary tract infections to life-threatening bacteremia and meningitis. For example, ExPEC is responsible for 80% of urinary tract infections and 30% of neonatal meningitis (Desvaux et al., 2020). These strains typically encode virulence factors that allow them to bind to eukaryotic cells (e.g., P-fimbriae, S-fimbriae), survive outside the intestines (e.g., siderophores), and damage cells and tissues (e.g., hemolysin, cytotoxic necrotizing factor) (Dale and Woodford, 2015).

Intestinal pathogenic *E. coli* strains are mucosal pathogens with six well-described diarrhea-associated pathotypes (i.e., enterotoxigenic *E. coli*, enterohemorrhagic *E. coli*, enteropathogenic *E. coli*, enteroinvasive *E. coli*, enteroaggregative *E. coli*, and diffusely adherent *E. coli*), which use different pathogenic strategies, such as the production of various toxins and host adhesion/invasion factors (Kaper et al., 2004). Intestinal pathogenic *E. coli* strains cause common food-borne diarrheal complications (Beutin and Martin, 2012; Tseng et al., 2014; Eppinger and Cebula, 2015; European Food Safety Authority [EFSA] and European Centre for Disease Prevention and Control, 2021), including acute infectious diarrhea in children seen in developing countries (Kosek et al., 2003; Dutta et al., 2013).

While pathogenic *E. coli* have been clearly identified as the causative agents of urogenital and diarrheal infections, the role of *E. coli* in other conditions, such as inflammatory bowel diseases (IBDs) and colorectal cancer, remains unclear (Tjalsma et al., 2012; Brennan and Garrett, 2016; Tilg et al., 2018; Wassenaar, 2018; Garrett, 2019; Chattopadhyay et al., 2021). Besides host-genetic and environmental factors, several bacteria have been found to be involved in the pathogenesis of different neoplastic conditions, and an abundance of *E. coli* has been found among these patients (Bonnet et al., 2014; Feng et al., 2015; Nakatsu et al., 2015). Reflecting this situation, *E. coli* strains with specific sets of virulence factors (e.g., adherent-invasive *E. coli*, colibactin-producing *E. coli*) are considered to be pathobionts rather than bacteria causing acute infection (Desvaux et al., 2020).

In our previous prospective study, we identified a higher prevalence of bacteriocin-producing strains among *E. coli* strains isolated from the biopsies of patients with current or previous colorectal neoplasia compared to *E. coli* from biopsies of healthy controls (Kohoutová et al., 2020). Since several bacteriocins can be considered virulence factors (reviewed in Bosák et al., 2021), this follow-up study aimed to characterize mucosal *E. coli* isolates for the presence of 35 genetic determinants encoding known virulence factors.

## Materials and methods

### Study design, cohort characterization, and ethical approval

This study extends our previous study, where the prevalence of bacteriocinogeny was used to characterize *E. coli* isolates from colorectal biopsies (Kohoutová et al., 2020). Here, *E. coli* isolates were classified into phylogenetic groups and further characterized with respect to the prevalence of 35 virulence determinants.

The biopsies (*n* = 187; up to 3 samples per individual) were collected from patients treated at the University Hospital, Hradec Králové (Czech Republic), between 2013 and 2017. All participants were Caucasians living in the Czech Republic. Out of 63 participants with colorectal neoplasia, 21 were classified as having non-advanced colorectal adenoma (nCRA), 20 with advanced colorectal adenoma (aCRA), and 22 with colorectal carcinoma (CRC). Advanced colorectal adenoma was defined as an adenoma with low-grade dysplasia and larger than 10 mm and/or high-grade dysplasia of any size and/or an adenoma of any size with a villous component (Mahajan et al., 2013). While 36 participants had current neoplasia, biopsies from 27 participants were collected after surgical or endoscopic removal of the neoplasm (an average of 56 months after removal, ranging between 1 and 164 months). For two patients, no *E. coli* isolate was collected (i.e., nCRA patient no. 9 and CRC patient no. 4; see below). Twenty healthy volunteers were enrolled as a control group (*n* = 52, up to 3 biopsies per individual). Control individuals had an average risk for colorectal carcinoma, normal colonoscopy findings, and no history of colorectal neoplasia or inflammatory bowel disease. Demographic and clinical characteristics of the participants, as well as information about bowel preparation followed by collection of bioptic samples have been published previously (Kohoutová et al., 2020). All cohorts were matched for age and sex (Kohoutová et al., 2020).

All human clinical samples were collected after receiving written informed consent from participants. All data used in the study were anonymized, and the study was approved by the Joint Ethics Committee (Charles University, Faculty of Medicine at Hradec Králové, and the University Teaching Hospital Hradec Králové; Protocol no. 201107S54).

### Isolation and identification of *Escherichia coli* strains

We used *E. coli* strains isolated from mucosal biopsies obtained in the previous study (Kohoutová et al., 2020). Briefly, mucosal biopsies were collected during diagnostic or therapeutic colonoscopy, and the bioptic samples were cultured on MacConkey agar plates. A set of 522 candidate colonies (1–5 per biopsy) have been analyzed using VITEK 2 system (BioMérieux SA, Marcy l’Etoile, France), which resulted in 317 isolates identified as *Escherichia coli*. Seventy-one duplicate *E. coli* isolates (i.e., isolates originating from the same individual, belonging to the same phylogroup, and having the same set of detected VAGs) were excluded. A set of *E. coli* isolates (*n* = 246) with different PCR profiles was further analyzed in this study, including 46 strains from healthy controls, 71 strains from patients with non-advanced colorectal adenoma (nCRA), 65 strains from patients with advanced colorectal adenoma (aCRA), and 64 strains from patients with colorectal cancer (CRC). In two patients, one nCRA and one CRC, no *E. coli* strains were isolated (Supplementary Table 1).

### Phylogenetic classification of *Escherichia coli* isolates

Multiplex-PCR amplifying of the *chuA*, *yjaA*, and the TspE4.C2 genomic fragments were used to classify *E. coli* isolates into four phylogenetic groups (i.e., A, B1, B2, and D) (Clermont et al., 2000).

### PCR detection of virulence-associated genes

Due to unclear role of *E. coli* in neoplasia, *E. coli* isolates have been screened for the presence of 35 virulence determinants, which are relevant for known intestinal and extraintestinal *E. coli* pathotypes, such as determinants associated with binding to the host cell (*afaI*, *bfpA*, *eaeA*, *fimA*, *pap*, pCVD432, *sfa*, and *tsh*), with iron acquisition (*eitA*, *etsA*, *fepC*, *fyuA*, *ireA*, *iroN*, *iucD*, and *sitA*), with damage caused to cells and tissues (α*-hly, cdt*, *cnf1*, *ehly*, *lt*, *pks*, *sat*, *st*, *stx*_1_, *stx*_2_, and *usp*), with invasion (*ibeA*, *ial*, and *ipaH*), and with protection of bacterial cell (*iss*, *hlyF*, *kpsMTII*, *ompT*, and *traT*).

The complete set used in this study included the following determinants: α*-hly* – α-hemolysin, *afaI* – afimbrial adhesin, *bfpA* – bundle-forming pilus, *cdt* – cytolethal distending toxin, *cnf1* – cytotoxic necrotizing factor, *eaeA* – intimin, *ehly* – enterohemolysin, *eitA* – iron transport, *etsA* – transport system, *fepC* – enterobactin transport, *fimA* – fimbriae type I, *fyuA* – yersiniabactin receptor, *hlyF* – overproduction of outer membrane vesicles, *ial* – locus associated with invasivity, *ibeA* – invasion of brain epithelium protein A, *ipaH* – locus associated with invasivity, *ireA* – iron responsive element, *iroN* – salmochelin receptor, *iss* – increased serum survival protein, *iucD* – aerobactin synthesis, *kpsMTII* – capsule synthesis, *lt* – thermolabile enterotoxin, *ompT* – outer membrane protease T, *pap* – P-fimbriae, pCVD432 – aggregative adherence plasmid, *pks* – colibactin synthesis, *sfa* – S-fimbriae, *sat* – secreted autotransporter toxin, *sitA* – iron transport, *st* – thermostable enterotoxin, *stx*_1_ – Shiga toxin 1, *stx*_2_ – Shiga toxin 2, *traT* – complement resistance protein, *tsh* – temperature-sensitive hemagglutinin, and *usp* – uropathogenic-specific protein. In PCR screening, appropriate positive control *E. coli* strain for each VAG was used. The complete list of primers and PCR profiles is shown in Supplementary Table 2.

### Statistical analysis

The two-tailed Fisher’s exact test was used to analyze the prevalence of the genetic determinants of the phylogenetic groups and virulence factors. *P*-values lower than 0.05 were considered statistically significant and are denoted with asterisks according to statistical significance (*p < 0.05, ^**^p < 0.01, and ^***^p < 0.001). In cases of multiple testing, statistical significance was adjusted for the false discovery rate (*p*_adj_ < 0.0125). GraphPad Prism 5 software was used for calculations. Correlation analysis (Pearson coefficient) was performed using R software (v4.2.0) (R Core Team, 2022).

## Results

### Characterization of mucosal *Escherichia coli* isolates

*E. coli* isolates used in this study were obtained from mucosal biopsies of patients with colorectal neoplasia (*n* = 61; *E. coli* was not isolated from two patients) and healthy controls (*n* = 20). From 239 biopsies, a set of 317 *E. coli* isolates has been collected. While fourteen isolates represented duplicate *E. coli* isolation (isolation of identical strains) from the same biopsy, seventy-one *E. coli* isolates were duplicates from the same individual (see Methods). For reduction of bias in statistical analysis, isolates identified as duplicates have been excluded from the study and the set of 246 different *E. coli* strains has been further analyzed. The source and characteristics for each *E. coli* isolate (including duplicate isolates) are shown in Supplementary Table 1.

**FIGURE 1:**
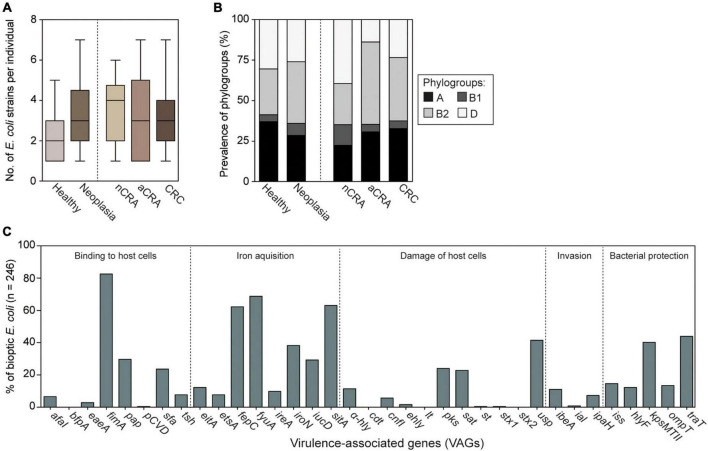
Collection and characterization of mucosal *E. coli* isolates from patients (*n* = 200) and healthy controls (*n* = 46). **(A)** On average, three various *E. coli* isolates were collected (range 1–7) per participant; there were no differences between healthy controls and neoplasia groups (Box and Whiskers: median, max/min). Lower numbers of *E. coli* isolates per healthy volunteer resulted from lower number of biopsies compared to the average number of biopsies per patient. **(B)** Prevalence of phylogroups was similar among *E. coli* from healthy controls and neoplasia patients. The phylogroup composition of *E. coli* strains differed between patients with advanced adenomas and the control group (*p* < 0.05, Fisher’s exact test, see Supplementary Table 3), but the difference was not statistically significant after correction for the false discovery rate (*p*_adj_ < 0.0125). **(C)** Prevalence of 35 VAGs has been determined in all *E. coli* isolates. The neoplasia group consisted of isolates from non-advanced colorectal adenoma (nCRA; *n* = 71), advanced colorectal adenoma (aCRA; *n* = 65), and colorectal carcinoma (CRC; *n* = 64).

Lower numbers of biopsies were collected from healthy individuals compared to patients (*p* < 0.05); however, the number of obtained *E. coli* isolates per biopsy was similar between patients and controls (*p* = 0.1335). On average, three different *E. coli* strains were collected for each participant (Figure 1A).

Phylogroup analysis of mucosal *E. coli* (*n* = 246) revealed phylogroups B2 (36.2%), A (30.1%), and D (26.8%) were common, while phylogroup B1 was relatively rare (6.9%). The *E. coli* phylogroups did not differ significantly between patients with neoplasia and healthy controls (*p* > 0.05, Figure 1B and Supplementary Table 3). The prevalence of *E. coli* from phylogroup B2 was higher among patients with advanced colorectal adenoma compared to healthy controls (*p* = 0.0201); however, this was not statistically significant after correction for the false discovery rate (*p*_adj_ < 0.0125, Figure 1B and Supplementary Table 3).

Analysis of the prevalence of 35 determinants encoding virulence factors showed that virulence genes associated with diarrheal *E. coli* pathotypes (i.e., *bfpA*, *ial*, pCVD432, *lt*, *st*, stx_1_, and stx_2_) were rarely found in the set of mucosal *E. coli*. At the same time, genes encoding fimbriae type 1 and three iron acquisition systems have been found in more than half of mucosal isolates (*fimA* 82.5%, *fyuA* 68.7%, *sitA* 63.0%, and *fepC* 62.2%). For more details see Figure 1C and Supplementary Table 3.

### Mucosal *Escherichia coli* from patients with neoplasia and healthy volunteers differ in the prevalence of virulence determinants

**FIGURE 2:**
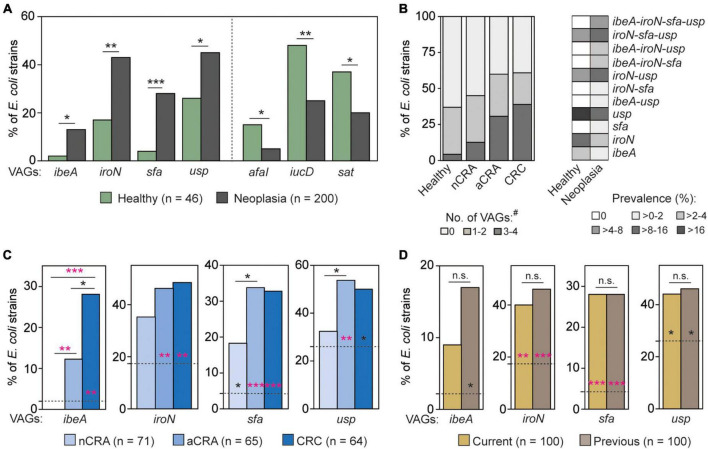
Virulence determinants with significantly different prevalence between mucosal *E. coli* from neoplasia patients and healthy controls. **(A)** Among *E. coli* from patients, the prevalence of four virulence-associated genes (VAGs) was higher (left), and the prevalence of three others was lower (right). **(B)** Certain combinations of neoplasia-associated genes (i.e., *ibeA*, *iroN*, *sfa*, and *usp*) occurred frequently in *E. coli* from patients, while *E. coli* from healthy controls harbored these VAGs individually. The VAGs with higher prevalence in the neoplasia group were mainly associated with *E. coli* from patients with advanced adenoma or carcinoma **(C)**, and the prevalence of these genes was not only higher in *E. coli* from current neoplasia patients but also in patients with a history of adenoma or carcinoma **(D)**. The two-tailed Fisher’s exact test was used to calculate the statistical significance between healthy controls and groups of patients (*p < 0.05, **p < 0.01, and ***p < 0.001). In panels **(C,D)**, the dotted lines represent the prevalence in *E. coli* from healthy controls, and the statistical significance relative to neoplasia stages is shown by asterisks. Statistical significance after correction for the false discovery rate (*p*_adj_ < 0.0125) is shown in pink. *E. coli* from patients with non-advanced colorectal adenoma (nCRA; *n* = 71), advanced colorectal adenoma (aCRA; *n* = 65), and colorectal carcinoma (CRC; *n* = 64) or patients with current and previous neoplasia (*n* = 100 and *n* = 100, respectively) represent subsets of neoplasia isolates (*n* = 200). # = the number of detected VAGs per isolate.

*E. coli* strains isolated from healthy controls and patients with neoplasia (i.e., nCRA, aCRA and CRC patients) differed in the prevalence of several virulence determinants (*p* < 0.05; Figure 2A). Besides the three determinants with lower prevalence among *E. coli* from patients (i.e., *afaI*, *iucD*, and *sat*), genes encoding S-fimbriae (*sfa*), siderophore receptor (*iroN*), invasin (*ibeA*), and genotoxin (*usp*) showed higher prevalence in *E. coli* from mucosal biopsies of neoplasia patients (Figure 2A).

More than half (55.0%) of *E. coli* isolates from neoplasia patients harbored at least one of the four abovementioned genes (i.e., *ibeA, iroN*, *sfa*, and *usp*). While *E. coli* from healthy controls often harbored them individually (e.g., *usp* in 17.4%, Figure 2B right), these genes co-occurred (co-occurrence was defined as the presence of 3 or 4 such genes) more frequently in *E. coli* from patients (27.0% vs. 4.3%, respectively; *p* < 0.001, Figure 2B). These four genes showed positive correlation with each other (*iroN*/*sfa* (*R* = 0.63), *usp*/*iroN* (*R* = 0.61), *usp*/*sfa* (*R* = 0.43), *iroN*/*ibeA* (*R* = 0.34), *ibeA*/*sfa* (*R* = 0.33), and *usp*/*ibeA* (*R* = 0.26); *p* < 0.001), as well as to advanced neoplasia stages (*p* < 0.05; see Supplementary Table 4). Combination *sfa*-*iroN*-*usp* has been the most frequent combination in *E. coli* from patients with advanced neoplasia (21.5% in aCRA and 12.5% in CRC). Moreover, combination of all four identified VAGs has been found exclusively in *E. coli* from patients with advanced neoplasia (9.2 and 10.9% in aCRA and CRC, respectively) (Supplementary Table 1). While some combinations of neoplasia-associated virulence genes were frequent, others were not found in our set of *E. coli* isolates (Figure 2B).

The prevalence of these four virulence genes also differed among *E. coli* from patients with various neoplasia stages. These genes were common in *E. coli* strains from patients with more advanced stages of neoplasia (*p* < 0.05; Figure 2C), including statistically significant differences after correction for testing of multiple groups (*p*_adj_ < 0.0125, Figure 2C). A higher prevalence of *ibeA, iroN*, *sfa*, and *usp* was also found in *E. coli* from patients with previous colorectal neoplasia (*p* < 0.05; Figure 2D).

On the other hand, the prevalence of three virulence genes, which were negatively-associated with *E. coli* from neoplasia (i.e., *afa*, *iucD*, and *sat*), did not differ among neoplasia stages. The different prevalence of *iucD* among *E. coli* from patients with current and previous neoplasia (*p* < 0.0332) was not statistically significant after correction for the false discovery rate (*p*_adj_ < 0.0125). A complete statistical analysis is shown in Supplementary Table 3.

### Identified virulence factors were common in individuals with neoplasia

**FIGURE 3:**
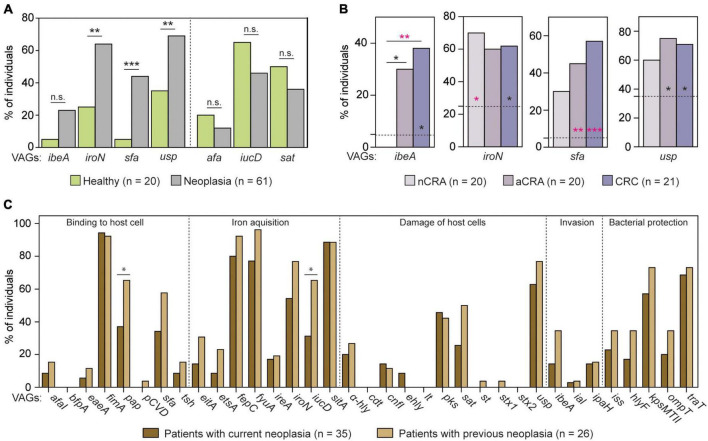
Prevalence of virulence determinants in patients and healthy individuals. **(A)** Out of 35 analyzed virulence-associated genes (VAGs), *iroN*, *sfa*, and *usp* were more frequent in neoplasia patients compared to healthy individuals. **(B)** In addition, higher prevalence of *ibeA*, *sfa*, and *usp* was found among patients with advanced neoplasia stages. **(C)** The most of the analyzed VAGs showed similar prevalence between patients with current and with previous neoplasia. The two-tailed Fisher’s exact test was used to calculate the statistical significance between healthy controls and groups of patients (*p < 0.05, **p < 0.01, and ***p < 0.001). In panel **(B)**, the dotted lines represent the prevalence in healthy controls, and asterisks show the statistical significance relative to neoplasia stages. Statistical significance after correction for the false discovery rate (*p*_adj_ < 0.0125) is shown in pink. The prevalence of VAGs was analyzed for each group of participants, i.e., healthy controls (HC, *n* = 20) and neoplasia patients [nCRA (*n* = 20), aCRA (*n* = 20), and CRC (*n* = 21)]. The complete analysis is shown in Supplementary Table 3.

Since different *E. coli* strains were collected from each participant (Figure 1A), we also analyzed the prevalence of thirty-five VAGs with respect to each individual. While a negative association between three determinants (*afaI*, *iucD*, and *sat*) and neoplasia was not found on the patient level, the prevalence of *iroN*, *sfa*, and *usp* was significantly higher among neoplasia patients compared to healthy controls (*p* < 0.05; Figure 3A and Supplementary Table 3). A non-significant increase in prevalence among patients was found for the *ibeA* gene (5.0 and 23.0%; *p* < 0.1000). At the same time, the *ibeA* gene was positively-associated with advanced neoplasia patients (*p* < 0.05; Figure 3B). This VAG was rare (<5%) among healthy controls and patients with non-advanced adenomas, but it was quite common among patients with advanced neoplasia stages (30.0 and 38.1%; *p* < 0.05 (aCRA) and *p*_adj_ < 0.0125 (CRC); Figure 3B and Supplementary Table 3). Prevalence of *ibeA* correlated with CRC diagnosis (*R* = 0.30, *p* < 0.01). In addition, the prevalence of *sfa* and *usp* was also higher in groups of patients with advanced neoplasia (*p* < 0.05; Figure 3B). Presence of all four VAGs (i.e., *ibeA, iroN*, *sfa*, and *usp*) in human intestines showed positive correlation with CRC patients (*R* = 0.29, *p* < 0.01), while border-line significance was found for negative correlation with controls and nCRA patients (*R* = -0.22, *p* = 0.054). Complete correlation analysis including graph of Correspondence analysis is shown in Supplementary Table 4.

Interestingly, patients with current (*n* = 35) and previous neoplasia (*n* = 26) showed similar prevalence of tested VAGs except of two determinants – *pap* (P-fimbriae) and *iucD* (aerobactin synthesis) (*p* < 0.05, Figure 3C).

## Discussion

Colorectal carcinoma is one of the most common human malignancies (Sung et al., 2021). Its etiopathogenesis has not been fully elucidated; however, recent studies have shown that the composition of the large intestine microbiota plays an important role in tumorigenesis, and *E. coli* is considered to be a bacterial species associated with colorectal neoplasia (Tjalsma et al., 2012; Bonnet et al., 2014; Feng et al., 2015; Brennan and Garrett, 2016; Tilg et al., 2018; Garrett, 2019; Chattopadhyay et al., 2021).

Our current study provided novel findings on the possible role of *E. coli* in colorectal neoplasia. Compared to other studies analyzing the role of *E. coli* at the microbiome level, we collected a comprehensive set of *E. coli* isolates and performed analyses at the *E. coli* strain and patient level. From each participant, several different sites in the intestines were biopsied and *E. coli* isolates were collected from each biopsy. This fact allowed analysis of prevalence of VAGs among (i) all collected *E. coli* isolates (*n* = 317), (ii) different isolates per biopsy (*n* = 303), (iii) different isolates per individual (*n* = 246), and (iv) the presence of VAGs in individuals (*n* = 81). Analysis of different *E. coli* per individual appears to be the most appropriate since the first two sets are potentially affected by the clonality of the collected *E. coli* isolates, and analysis on the patient level does not provide information about the occurrence of VAGs in bacterial cells. On average, three different *E. coli* strains were collected and characterized per individual. Based on the studies showing that the *E. coli* population typically consists of 1–3 different strains (Micenková et al., 2016a), a substantial part of participants’ *E. coli* strains were analyzed. In addition, differences in the prevalence of VAGs among *E. coli* strains were also found at the patient level indicating their relevance to patients’ health. Analysis performed with all collected *E. coli* isolates (*n* = 317) has been consistent with presented data (data not shown), suggesting only a minimal effect of potential duplicate isolates.

We determined the prevalence of 35 virulence-associated genes in *E. coli* strains obtained from three groups of patients diagnosed with colorectal neoplasia (i.e., non-advanced adenoma, advanced adenoma, and colorectal carcinoma) and a group of healthy controls. A positive association between virulence genes and neoplasia was predominantly found in patients with advanced adenoma and colorectal carcinoma (Figure 2C). *E. coli* from patients with non-advanced adenoma differed from *E. coli* of healthy controls and patients with advanced adenoma/carcinoma in several tested determinants, including virulence factors and *E. coli* phylogroups (Supplementary Table 3). As we showed in previous study, non-advanced adenomas were histologically different from both the healthy tissue and advanced adenomas (Kohoutová et al., 2018). Moreover, differences in intestinal microbiota in the early stages of neoplasia were shown by Nakatsu et al. (2015). On the other hand, it is also possible that some patients diagnosed with non-advanced colorectal adenoma could have had pathological conditions other than the first stage of colorectal carcinoma. Bonnington and Rutter (2016) showed that not all patients with colon adenomas have a higher risk of carcinoma than the general population.

In the neoplasia group, around 50% of patients had a neoplasm at the time of colonoscopy, while the other half had their neoplasm removed, on average 4.6 years before the current colonoscopy. Interestingly, the prevalence of *E. coli* phylogroups and the tested VAGs was similar in both neoplasia groups (Supplementary Table 3), suggesting that successful treatment of colorectal neoplasia does not result in a significant change in the intestinal *E. coli* population. The presence of particular *E. coli* strains in the gut is therefore not affected by the presence of colorectal neoplasia but rather implies the opposite scenario, i.e., the presence of particular *E. coli* strains is suspected of contributing to the development of colorectal neoplasia. In fact, the observed trend for higher occurrence of various VAGs (statistically significant for *pap* and *iucD*) in the group of patients with previous neoplasia (Figure 3C) further suggests the role of *E. coli* strains in tumorigenesis.

In this study, mucosal *E. coli* isolates frequently belonged to phylogroup B2; this was especially true for the advanced-adenoma and carcinoma groups. However, the prevalence of B2-isolates did not significantly differ between patients and healthy controls, which is in contrast to other colorectal cancer studies, where phylogroup B2 was associated with neoplasia (Kohoutová et al., 2014; Raisch et al., 2014; Nouri et al., 2021). The limited number of samples in this study likely precluded reaching statistical significance. *E. coli* strains of phylogroup B2 range from normal human resident microflora (Picard et al., 1999; Escobar-Páramo et al., 2004; Nowrouzian et al., 2005; Micenková et al., 2016a) to important human pathogens (Kotlowski et al., 2007; Azpiroz et al., 2009; Raisch et al., 2014; Micenková et al., 2016b), depending on encoded virulence factors.

We observed a trend toward a higher occurrence of *E. coli* strains producing colibactin (based on detected *clbB* gene) in patients with neoplasia compared to controls, although the significance was borderline (26.5 and 13.0%, *p* = 0.0573; Supplementary Table 3). Colibactin is genotoxin with a known role in carcinogenesis (Lopez et al., 2021), and its prevalence is frequently found to be higher among patients with colorectal cancer (Arthur et al., 2012; Buc et al., 2013; Sarshar et al., 2017). Compared to these studies, our set of participants combined patients with current- as well as previous neoplasia; however, *E. coli* from both groups showed similar prevalence of *pks* (29.0 and 24.0%, respectively; *p* = 0.5218). Thus, the reduced statistical significance of *pks* prevalence is more likely due to a small set of samples. Similarly, several other studies also failed to find a significantly higher prevalence of *pks* in patients with neoplasia (Shimpoh et al., 2017; Iwasaki et al., 2022), which suggests that additional factors also contribute to the development of colorectal neoplasia.

In contrast to colibactin, we identified a strong association between several other tested virulence factors and neoplasia. For the first time, a genetic determinant for uropathogenic-specific protein (*usp*) was shown to be associated with neoplasia. The uropathogenic-specific protein belongs to the group of bacteriocin-like proteins (Parret and De Mot, 2002). Interestingly, several bacteriocins could act as virulence factors (Bosák et al., 2021), and previously, we found increased production of bacteriocins in the patients with colorectal neoplasia (Kohoutová et al., 2014, 2020). Usp is frequently found in *E. coli* strains causing extraintestinal infections (Yamamoto et al., 2001; Rijavec et al., 2008) and in patients with inflammatory bowel diseases (Sepehri et al., 2011; Roche-Lima et al., 2018). Since Usp is a genotoxin active against mammalian cells (Nipič et al., 2013), presence of *E. coli* synthesizing this protein in the host intestines could contribute to inflammation and neoplasia.

Three additional determinants were positively-associated with neoplasia; they encoded S-fimbriae (*sfa*), siderophore receptor (*iroN*), and invasion of brain epithelium protein A (*ibeA*). All of them are known as virulence factors of extraintestinal pathogenic *E. coli* strains. S-fimbriae form structural adhesive organelles on the bacterial envelope facilitating adhesion to mammalian cells (Mulvey, 2002). Outer membrane protein IroN serves as a receptor for salmochelin (Hantke et al., 2003) and could contribute to the eukaryotic invasion of pathogenic *E. coli* (Feldmann et al., 2007), which was also shown for other siderophore receptors (Russo et al., 2001). Both virulence factors (i.e., S-fimbriae and salmochelin receptor) are frequently encoded on the same pathogenicity island (Dobrindt et al., 2001). Invasin IbeA is associated with pathogenic strains causing meningitis (Zhao et al., 2018) and is also responsible for the invasion of adherent-invasive *E. coli* (AIEC) into intestinal epithelium (Cieza et al., 2015). AIEC strains harboring *ibeA* are frequently found in patients with inflammatory bowel diseases (Martinez-Medina et al., 2009; Palmela et al., 2018), and Sarshar et al. (2017) found that around 20% of *E. coli* isolates taken from adenomatous polyps were positive for the *ibeA* gene. We clearly showed an association between the *ibeA* gene and neoplasia, including a positive correlation with neoplasia progression. In addition, the higher prevalence of AIEC strains in these pathologies could partially explain the increased risk of neoplasia in patients with inflammatory bowel diseases.

On the other hand, we found three genes [i.e., afimbrial adhesin (*afaI*), aerobactin synthesis (*iuc*D), and secreted autotransporter toxin (*sat*)] with significantly lower prevalence in neoplasia patients (Figure 2A). These VAGs are frequently found among various *E. coli* strains (Micenková et al., 2016b; Vieira et al., 2020). In contrast to our study, Prorok-Hamon et al. (2014) found increased incidence of *afaI* in patients with inflammatory bowel diseases and colon cancer. Afimbrial adhesin is a virulence factor typical for mucosa-associated *E. coli* pathotypes (e.g., DAEC and AIEC). Moreover, EPEC pathotype (encoding *eae*) has been found to be also associated with colon cancer (Maddocks et al., 2009; Viljoen et al., 2015), but prevalence of intimin gene (*eaeA*) was low in our set of *E. coli* from neoplasia patients (3%, Supplementary Table 3). We hypothesize that the observed lower prevalence of some virulence genes in neoplasia is a result of presence of other neoplasia-associated genes, having similar functions and being able to complement the absent VAGs, e.g., adhesion can be mediated by both *sfa* and *afaI*, iron acquisition by both *iroN* and *iucD*, and toxicity by both *usp* and *sat*.

About half of *E. coli* strains from neoplasia patients harbored at least one determinant (i.e., *ibeA, iroN*, *sfa*, and *usp*), and these virulence factors with activity against eukaryotic cells co-occurred frequently in specific combinations among *E. coli* strains from neoplasia patients. The same *E. coli* strains also often harbored genes for bacteriocins [86.4% compared to 52.2% of strains without the abovementioned virulence factors; based on the bacteriocin prevalence data published by Kohoutová et al. (2020)]. Interestingly, some bacteriocins are active also against eukaryotic cells (Bosák et al., 2021). These findings suggest that *E. coli* virulence in neoplasia is based on a combination of several virulence factors, likely representing a functional unit rather than being mediated by a single virulence factor or the sum of independent virulence factors. This has already been suggested for virulent *E. coli* strains causing different types of infection (Kaper et al., 2004). It is tempting to speculate that *E. coli*, as a pathobiont, has to harbor certain combinations of VAGs, which could participate in binding to the host cell (e.g., *sfa*/*iroN*), in invasion into host tissue (e.g., *ibeA/iroN*) and in damage to the host cell (e.g., *usp*). Long-term colonization of host intestines by these *E. coli* strains could result in a chronic inflammation and in development of colorectal neoplasia.

In conclusion, this study found a positive association between four virulence determinants of *E. coli* and colorectal neoplasia. These virulence-associated genes were predominantly found among *E. coli* from patients with advanced adenoma or colorectal carcinoma. The relevance of the observed associations is supported by statistical significance at the level of cohort patients as well as after correction for the false discovery rate. Since the identified virulence factors are involved in genotoxicity and adhesion/invasion process, *E. coli* virulence in neoplasia appears to include a combination of several virulence factors targeting intestinal epithelial cells that can contribute to chronic inflammation and neoplasia. Moreover, these strains stably colonize the gut, as suggested by the fact that treatment of colorectal neoplasia did not result in a significant decrease in their occurrence in the gut. Based on this, the presence of specific *E. coli* strains in the intestinal microflora appears to be a cause rather than a consequence of colorectal neoplasia.

## Data availability statement

The original contributions presented in this study are included in the article/Supplementary material, further inquiries can be directed to the corresponding author.

## Ethics statement

The studies involving human participants were reviewed and approved by Charles University, Faculty of Medicine at Hradec Králové, and the University Teaching Hospital Hradec Králové; Protocol no. 201107S54. The patients/participants provided their written informed consent to participate in this study.

## Author contributions

JBo and DŠ: study conception and design, manuscript writing. DK, MH, JK, PM, and SR: material preparation and data collection. JBo, DK, and DŠ: data analysis. JBo, DK, MH, JBu, and DŠ: review of the manuscript. All authors contributed to the article and approved the submitted version.

## Supplementary material

Supplementary Table 1 Characteristics of *Escherichia coli* isolates.

Supplementary Table 2 List of primers and PCR profiles.

Supplementary Table 3 Statistical analysis.

Supplementary Table 4 Correlation analysis.
